# Direct Mechanical Thrombectomy Versus Prior Bridging Intravenous Thrombolysis in Acute Ischemic Stroke: A Systematic Review and Meta-Analysis

**DOI:** 10.3390/life13010185

**Published:** 2023-01-09

**Authors:** Zahra Kolahchi, Nasrin Rahimian, Sara Momtazmanesh, Anahid Hamidianjahromi, Shima Shahjouei, Ashkan Mowla

**Affiliations:** 1 School of Medicine, Tehran University of Medical Sciences, Tehran 1417613151, Iran; 2 Department of Neurology, Creighton University Medical Center, Omaha, NE 68124, USA; 3 Department of Neurology, Feinberg School of Medicine, Northwestern University, Chicago, IL 60611, USA; 4 Department of Neurology, Barrow Neurological Institute, St. Joseph’s Hospital and Medical Center, Phoenix, AZ 85013, USA; 5 Division of Stroke and Endovascular Neurosurgery, Department of Neurological Surgery, Keck School of Medicine, University of Southern California, Los Angeles, CA 90033, USA

**Keywords:** acute ischemic stroke, mechanical thrombectomy, bridging therapy, intravenous thrombolysis

## Abstract

Background: The current guideline recommends using an intravenous tissue-type plasminogen activator (IV tPA) prior to mechanical thrombectomy (MT) in eligible acute ischemic stroke (AIS) with emergent large vessel occlusion (ELVO). Some recent studies found no significant differences in the long-term functional outcomes between bridging therapy (BT, i.e., IV tPA prior to MT) and direct MT (dMT). Methods: We conducted a systematic review and meta-analysis to compare the safety and functional outcomes between BT and dMT in AIS patients with ELVO who were eligible for IV tPA administration. Based on the ELVO location, patients were categorized as the anterior group (occlusion of the anterior circulation), or the combined group (occlusion of the anterior and/or posterior circulation). A subgroup analysis was performed based on the study type, i.e., RCT and non-RCT. Results: Thirteen studies (3985 patients) matched the eligibility criteria. Comparing the BT and dMT groups, no significant differences in terms of mortality and good functional outcome were observed at 90 days. Symptomatic intracranial hemorrhagic (sICH) events were more frequent in BT patients in the combined group (OR = 0.73, *p* = 0.02); this result remained significant only in the non-RCT subgroup (OR = 0.67, *p* = 0.03). The RCT subgroup had a significantly higher rate of successful revascularization in BT patients (OR = 0.73, *p* = 0.02). Conclusions: Our meta-analysis uncovered no significant differences in functional outcome and mortality rate at 90 days between dMT and BT in patients with AIS who had ELVO. Although BT performed better in terms of successful recanalization rate, there is a risk of increased sICH rate in this group.

## 1. Introduction

Stroke is the major cause of long-term neurological deficits in people and the second greatest cause of mortality globally [1]. Between the 1970s and the early 2000s, stroke incidence significantly declined in high-income nations due to the greater use of preventative therapies and large drops in risk factors [2]. Studies show that the incidence of stroke has been decreasing in high-income countries in the past 30 years too [3]. However, even if age-specific stroke incidence continues to decline at its current rate, the overall number of new stroke patients each year in high-income nations would keep rising during the following 30 years, due to the aging population [3]. Almost 91% of strokes in high-income nations are ischemic type [4]. Choosing the proper therapy, improving treatment effectiveness, increasing people’s knowledge of stroke risk factors and warning signs and reducing the time between symptom onset and admission, can decrease the mortality and morbidity of stroke patients [5].

Emergent large vessel occlusion (ELVO) accounts for 24 to 46% of acute ischemic strokes (AIS) [6]. Compared to non-LVO strokes, ischemic strokes caused by ELVOs have bigger infarct sizes [7,8], more severe symptoms [9,10], and worse long-term prognosis [11,12]. ELVOs usually involve proximal vessels, so crucial brain areas are affected, leading to significant neurological deficits [13]. According to prior studies, individuals with ELVOs had higher rates of dependency or mortality at 3 to 6 months after AIS (measured by a modified Rankin Scale (mRS) score of 3–6) compared with patients without ELVOs [14]. Additionally, ELVO AIS patients had considerably higher six-month death rates than non-ELVO AIS patients [14]. Hence, ELVO ischemic strokes are expected to have a greater impact on poststroke dependency and mortality on the populations’ health than they do on the incidence rate in acutely presenting individuals.

Considering the reliable results of mechanical thrombectomy (MT), the management of AIS is one of the areas that is advancing the fastest. Up until the middle of the 1990s, stroke therapy had made very little to no progress. It was discovered that intravenous tissue plasminogen activator (IV tPA) had some (but limited) advantages [15]. However, due to the small treatment window (originally 3 hours, then increased to 4.5 hours) where only 3.4–5.2% of stroke patients receive IV tPA [16], the low rate of vessel recanalization (13–50%) [17,18,19,20], and the low rate of favorable outcomes (12.9–30%) [21,22], considerable effort has been made to achieve outcomes comparable to or equal to those in the cardiac field.

In 2013, three randomized controlled trials (RCTs) investigating catheter-based stroke treatments for ELVO failed to provide any discernible improvement [23,24,25]. Around two years later, five RCTs from various nations found that MT for AIS delivered overwhelmingly beneficial outcomes, which led to a fundamental change in AIS management [26,27,28,29,30].

The standard treatment for AIS due to ELVO is IV tPA followed by MT (bridging therapy, BT) if patients have the eligibility criteria [31]. However, there is still uncertainty around the superiority of BT over direct mechanical thrombectomy (dMT) in these patients. Although some studies suggested that IV tPA may increase the downstream perfusion of large occluded arteries and improve the ischemic stroke outcomes [32], the recanalization rate is relatively low [33,34]. Others have shown no significant difference in terms of functional outcome between patients who received BT versus those treated with dMT [35].

Moreover, IV tPA may increase the chance of intracranial hemorrhage [36,37,38], thrombus fragmentation and migration, that might reduce the rate of reperfusion and complicate MT [39,40]. Observational data from single-center series, as well as pooled and meta-analyses, suggested that dMT can be as effective as BT in ELVO patients [41,42,43]. Four recent RCTs found that BT was not superior to dMT in stroke patients [44,45,46,47]. We aimed this systematic review and meta-analysis to throw some light on the functional outcome of BT compared to dMT, based on the latest literature.

## 2. Methods

This meta-analysis followed the PRISMA (Preferred reporting items for systematic reviews and meta-analyses) guidelines [48]. The authors declare that all supporting data are available within the article and in the Data Supplement. If certain data is requested, the corresponding author will provide the additional data.

### 2.1. Search Strategy

The electronic databases of PubMed, EMBASE, Scopus, and Cochrane Library (up to November 2021) were systematically searched using the titles and the abstracts retrieval method with no language restrictions. We obtained the selected keywords by reviewing primary search results, experts’ opinions, and controlled vocabularies—medical subject headings (MeSH) in Pubmed and Excerpta Medica Tree (EMTREE) in Embase. Forward and backward citation tracking of the identified included articles was also conducted to reveal further relevant studies. Details of search strategies are available in the Data Supplement (Table S1).

### 2.2. Eligibility Criteria

The included articles met all the following inclusion criteria: (1) studies that investigated patients with AIS in a large vessel territory—including M1 or M2 segments of the middle cerebral artery (MCA), terminal internal carotid artery (ICA), posterior cerebral artery, or basilar artery; (2) studies that compared outcomes for dMT with BT; (3) patients eligible for IV tPA in both groups (dMT and BT); (4) the desired mechanical thrombectomy strategies in this study were direct aspiration, stent retriever thrombectomy, or stent retrieval under aspiration. Of note, the majority of these studies were published after 2011.

Excluded articles had the following criteria: (1) intraarterial thrombolysis, (2) AIS without ELVO.

Case reports, commentary, conference abstracts, editorials, letters, and reviews were excluded. If duplicate studies were identified, those with a larger sample of patients, or more recent publication were included.

### 2.3. Outcome Definition

The primary outcome endpoint was good functional outcome used to assess the efficacy of BT compared to dMT, defined as an mRS score of 0–2 at three months. The secondary outcomes were mortality within 90 days, successful recanalization—defined as thrombolysis in cerebral infarction scores (TICI) 2b to 3, and safety outcome was defined as symptomatic intracranial hemorrhage (sICH), regardless of the guideline used for its definition.

### 2.4. Data Extraction and Assessment

Two researchers reviewed the titles and abstracts and screened the candidate articles, extracted the data per provided protocol, and reached an agreement on all items by discussion if necessary. The data extraction protocol included baseline characteristics, primary outcomes, and secondary outcomes.

### 2.5. Quality Evaluation of the Included Studies

For quality assessment of the included articles, we used ROBINS-I (risk of bias in non-randomized studies of interventions) and ROB-2 for quality evaluation of the enrolled observational studies and RCTs, respectively. The risk of bias assessment outcome was defined as low, moderate, or serious (high). Any conflicts were resolved by discussion or consultation with a third reviewer.

### 2.6. Statistical Analysis

Odds ratio (OR) with 95% confidence interval (CI) was used as the measure of the effect for comparison of each outcome between patients receiving dMT and those with BT. The SMD of ≤0.2, 0.2–0.8, and 0.8 ≤ represented small, moderate, and large effect sizes. We used Cochran’s Q test and the I2 index to assess heterogeneity between studies in the between-group meta-analyses. The I2 indices of ≤25, 26–75, and 75% ≤ represented low, moderate, and high degrees of heterogeneity, respectively [49]. We used fixed effects models as the results were homogeneous (I2 < 40% and *p* > 0.05). Random effect models according to the DerSimonian and Laird method [50] were planned to be used if the results were otherwise [51]. We categorized the included studies into two groups (anterior and combined). The anterior group included studies with large vessel occlusion in anterior circulation only, while the combined group included studies with either anterior or posterior circulation. To reduce the heterogeneity between individual studies, we also performed subgroup analyses based on the study type (RCT versus non-RCT). Publication bias was initially assessed by visual observation of the degree of funnel plot asymmetry. Then, we used Egger’s bias test [52] to confirm the visual perception from the funnel plot objectively. A *p*-value < 0.1 was considered evidence of publication bias. The funnel is available in the supplementary material. All statistics were performed using “meta” (version 4.17-0), “metafor” (version 2.4-0), and "dmetar" (version 0.0-9) packages, R (R Core Team (2020). R: A language and environment for statistical computing. R Foundation for Statistical Computing, Vienna, Austria) and STATA. A *p*-value of <0.05 was considered statistically significant.

## 3. Results

**Figure 1:**
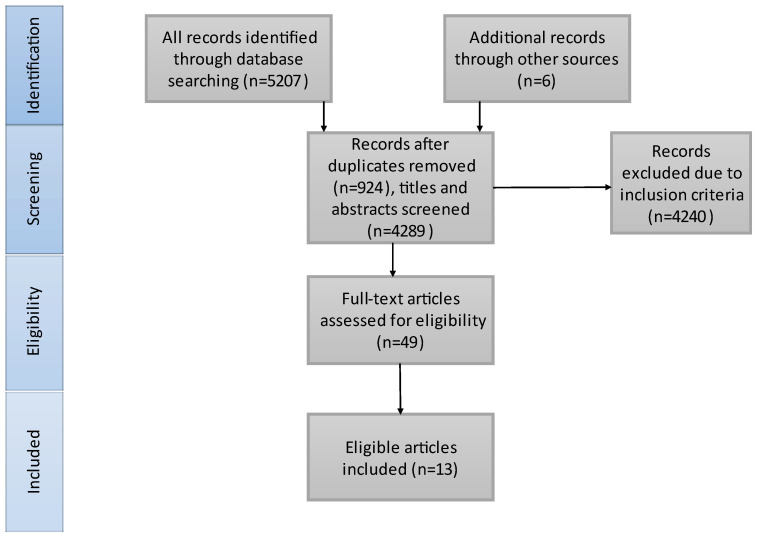
PRISMA flowchart of the search strategy and study selection. On the basis of our search strategy and also articles from other sources, we found 4289 records. After excluding irrelevant articles and full-text screening, 13 articles were identified for inclusion in our systematic review.

The literature search results are presented in Figure 1. We included 3985 patients in our study, 2113 received BT treatment and 1872 underwent dMT. Thirteen studies matched our criteria and were included in our meta-analysis. A total of 10/13 of the included studies contained only anterior circulation AIS patients. Bellwald et al. [35] presented the patients with AIS in the anterior circulation from Broeg-Morvay A. et al. [42] and Weber R. et al. [41]. Thus, we considered Bellwald et al. representative of the anterior group and the other two [41,42] for the combined group. Four of the included articles are RCTs. Table 1 illustrates the characteristics of the included studies and Table 2 shows the baseline characteristics of the included patients. SICH criteria were different in the included studies: European Cooperative Acute Stroke Study (ECASS) III [35,41,44], ECASS II [44,53], Heidelberg Bleeding Classification [45,47,54,55], Safe Implementation of Thrombolysis in Stroke-Monitoring Study (SITS-MOST) [44,46] and National Institute of Neurological Disorders and Stroke (NINDS) [44].

The risk of bias assessment outcome is presented in Figures S2 and S3.

### 3.1. Mortality at 90 Days

**Figure 2:**
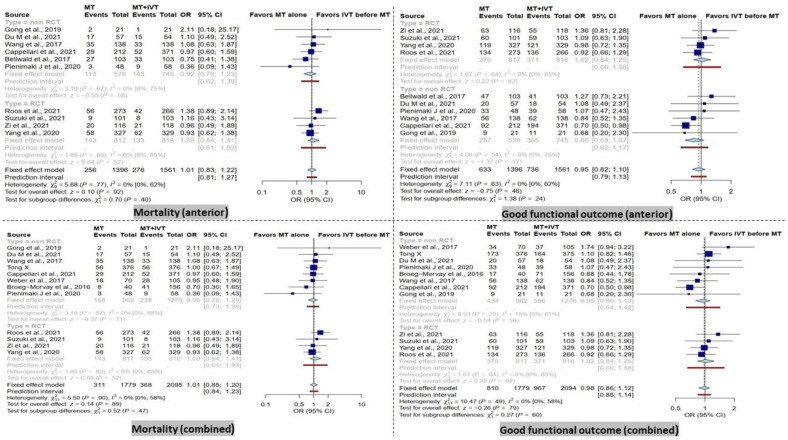
Forest plots of comparison of mortality and good functional outcome (modified Rankin Scale ≤ 2) at 90 days between direct mechanical thrombectomy and bridge therapy in anterior and combined groups [35,41,42,44,45,46,47,53,55,56,57,58,59].

In the combined group, there was no significant difference between dMT patients compared to BT patients in terms of mortality within 90 days. Furthermore, the subgroup analysis in RCT and non-RCT studies comparing dMT versus BT patients did not reveal any significant differences in the combined group (Figure 2).

Furthermore, no significant difference was observed between dMT patients compared to BT patients in the anterior group in terms of mortality within 90 days. These results remained similar in the subgroup analysis of RCT and non-RCT type studies (Figure 2).

### 3.2. Functional Outcome at 90 Days

There was no significant difference in terms of good functional outcome (mRS score ≤ 2) at 90 days between patients receiving dMT and those with BT in the combined analysis. Correspondingly, the subgroup analysis showed a non-significant difference in the RCT and non-RCT subgroups (Figure 2).

In addition, no significant difference in good functional outcome (mRS score ≤ 2) at 90 days was detected in the anterior group in RCT and non-RCT subgroups (Figure 2).

### 3.3. sICH

BT patients had significantly higher rates of sICH in the combined group (OR = 0.73 [95% CI, 0.56–0.96], *I*^2^ = 0%, *p* = 0.02). After subgroup analysis based on study type, this result remained significant only in the non-RCT subgroup (OR = 0.67 [95% CI, 0.46–0.96], *I*^2^ = 0%, *p* = 0.03).

**Figure 3:**
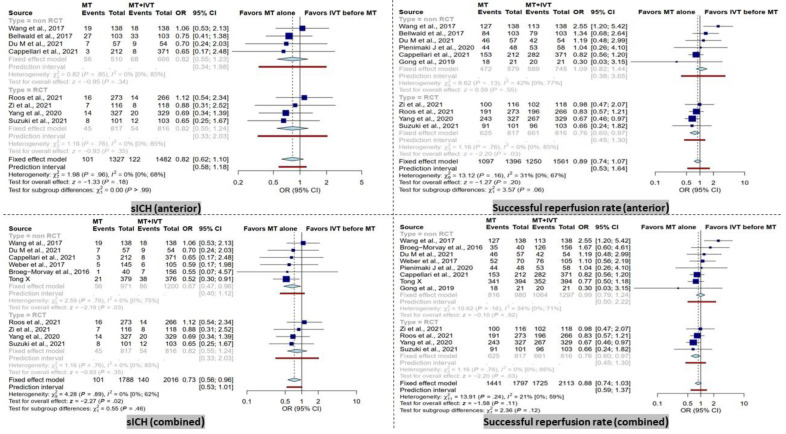
Forest plots of comparison of symptomatic intracranial hemorrhage (sICH) events and successful reperfusion rate (TICI 2b-3) between direct mechanical thrombectomy and bridge therapy in anterior and combined groups. TICI: thrombolysis in cerebral infarction [35,41,42,44,45,46,47,53,56,57,58,59].

In contrast, within the anterior group analysis, no significant difference was observed. Furthermore, the subgroup analysis of RCT and non-RCT studies did not reveal any significant difference (Figure 3).

### 3.4. Successful Recanalization

No significant difference in successful recanalization rate (TICI score 2b-3) was observed in the combined group (Figure 3). However, the RCT subgroup showed a significantly higher successful recanalization rate in BT patients (OR = 0.73 [95% CI, 0.56–0.96], *I*^2^ = 0%, *p* = 0.02).

In the anterior group, comparable results were observed in terms of successful recanalization rate when comparing dMT patients with the BT group. Only in the RCT subgroup, a significantly higher successful recanalization rate in BT patients was noted (OR = 0.73 [95% CI, 0.56–0.96], *I*^2^ = 0%, *p* = 0.02). 

## 4. Discussion

The present systematic review and meta-analysis included 13 RCT and observational studies on IV tPA eligible patients in both dMT and BT groups. We excluded studies including patients who were not eligible for IV tPA. We observed that dMT, as compared to BT, may be similarly efficacious but might result in a reduced risk of sICH occurring in patients with AIS. There was no significant difference in mortality and good functional outcome (MRS ≤ 2) at 90 days between dMT group and BT group, even after the subgroup analysis considering the RCT and non-RCT studies.

The safety and effectiveness of administrating IV tPA prior to MT for patients with AIS is still a debatable matter. Although the current guideline recommends using the IV tPA prior to MT (BT) [31], some recent RCTs and observational studies found no significant difference in functional outcomes between patients receiving BT and dMT [35,42,44,45,53,55,56,58,59]. Furthermore, a lower rate of sICH in favor of dMT has been reported [42,53,55,59]. In contrast, the two recent RCTs were unable to show non-inferiority of dMT with regard to functional outcome [46,47]. Of note, a valid comparison of functional outcomes between dMT and BT groups could be made only if all patients in both groups met the criteria for IV tPA administration. Obviously, including IV tPA-ineligible patients, such as patients taking anti-coagulants or with other contraindications [60,61,62,63,64,65,66] in the dMT group might result in poorer outcomes and affect the final comparisons [35].

ELVOs emerge through four different mechanisms, intracranial atherosclerosis that then developed into occlusion of an artery, plaque rupture or atherosclerotic embolism from extracranial arteries, cardioembolic causes, and cryptogenic stroke [67]. When blood flow toward the brain parenchyma is inadequate, ELVOs typically lead to cellular bioenergetic impairment and inflammatory responses that terminate with the death of neurons, glia, as well as endothelial cells [68]. Despite the fact that ischemic alterations take place within minutes, the severity and duration of hypoperfusion ultimately define the amount of infarcted tissue [68]; also, the degree of collateral blood flow to an ischemic location has a significant role in stroke development. Accordingly, one study reported that over 15% of transient ischemic attack (TIA) patients were discovered to have underlying ELVOs [12]; the absence of neurological symptoms or strokes in these cases was most likely caused by sufficient collateral perfusion.

There is an assumption that IV tPA prior to MT softens the thrombus and might improve the likelihood of successful recanalization. Since IV tPA accelerates the degradation of fibrin polymers, a smaller size of clot is anticipated in patients receiving BT [69]. IV tPA might also not entirely dissolve a big clot, but it may detach the embolus’ surface, making retrieval and aspiration simpler. Following MT, periprocedural thrombus debris is frequent and might result in downstream microvascular occlusions, which prevents reperfusion of distal arteries [70,71]. Early administration of alteplase results in an immediate and noticeable hypofibrinogenemia that inhibits platelet aggregation and dissolves loose platelet aggregations, maintaining the patency of downstream microvascular thrombosis [32]. None of the included studies in our meta-analysis reported a higher rate of successful recanalization in the BT group. In addition, Wang et al. [59] observed a significantly lower successful recanalization rate in patients treated by BT. Interestingly, our meta-analysis showed that there is no significant change in successful recanalization rate between the dMT and BT groups; however, the subanalysis of RCT studies, including 1633 patients, revealed a significantly higher successful recanalization rate in the BT group. It has been shown consistently that the sooner recanalization is achieved, the better the outcome will be [72]. The processes of counseling patients or next of kin, obtaining informed consent, drug preparation and administration take time prior to IV tPA and prolong the door to puncture time in patients receiving BT [59]. The door to puncture time was significantly different between BT and dMT in several studies [35,41,53,55,57,59], while others showed no difference [44,46,56]. The preparation time for IV tPA and the interval between IV tPA administration and groin puncture are important factors in determining door to puncture time in the BT group [46,55].

The rate of successful revascularization after IV tPA and prior to MT is shown to be relatively low in previous studies (11–20%) [33,73]. Successful recanalization with IV tPA has been shown to be associated with several factors including the distal occlusion site of thrombus and thrombus being partially occlusive [33,73,74,75]. Thus, BT might be a better choice for selected groups of patients [33].

Although higher rates of recanalization have been associated with better functional outcome and lower mortality in prior studies [76], our study showed that there was no significant difference in the rate of good functional outcome (mRS ≤ 2) and mortality within 90 days between dMT and BT in anterior and combined groups and also in the subgroup analysis. Similar to our study, Zhang et al. [77] compared dMT with BT in IV tPA eligible patients with ELVO. The results of their meta-analysis (nine studies including three RCTs and six observational studies) showed no differences in the rates of successful recanalization and mortality at 90 days. The rate of sICH was less common in the dMT group compared with the BT group in unadjusted analysis. The adjusted (for baseline characteristics) analysis showed no difference in the rate of sICH between the two groups, the dMT group showed a lower risk of any ICH compared to the BT group.

sICH is a well-known side effect of IV tPA [78,79,80,81]. Our study revealed that the sICH rate was significantly increased in the combined group and also in the non-RCT studies subgroup in the combined group. The anterior and RCT subgroup did not show any significant difference in terms of sICH rates between BT and dMT groups. The underlying mechanism of intracerebral hemorrhage is vascular disruption [82]. Although it has been shown that MT itself can disrupt the vessel wall, the use of IV tPA before MT might increase the risk of sICH associated with vessel wall damage. Plasminogen activators, which are used to dissolve clots, can accelerate the damage to the vasculature, and increase the blood–brain barrier (BBB) permeability through a variety of processes, including the production of metalloproteinases by interactions with particular endothelial receptors [83,84,85]. While our study showed no difference in mortality and clinical outcome in 90 days between BT and dMT groups, previous studies have demonstrated that ICH and sICH are associated with poorer prognosis [86,87,88]. This finding might be explained by the contrast agent’s cytotoxic impact and the mass effect generated by the loss of BBB, which compresses the nearby healthy tissue, impairing functional recovery [88].

Our study has several limitations. First, we did not consider the IV tPA dose in our analysis. The majority of the studies used the standard IV tPA dosage, which is 0.9 mg/kg. However, one study [46] administered 0.6 mg/kg and two studies [56,57] did not mention the IV tPA dosage. Second, we tried to minimize the impact of clot location by categorizing the patients in combined and anterior groups, but we could not conduct the subgroup analysis based on the exact occlusion site. Distal ELVOs may respond better to IV tPA when compared to proximal ones, and this difference may influence the results [33].

## 5. Conclusions

Our meta-analysis demonstrated that there is no significant difference in good functional outcome (mRS score ≤ 2) and mortality rate at 90 days between dMT and BT in patients with ELVO acute ischemic strokes. Although our results showed that BT performed better in terms of successful recanalization rate (TICI score 2b-3), we found an increased rate of sICH in BT patients.

## Figures and Tables

**Table 1:** Characteristics of included studies. mg: milligram; kg: kilogram; RCT: randomized controlled trial; ICA: internal carotid artery; M1: M1 segment of the middle cerebral artery; M2: M2 segment of the middle cerebral artery; MCA: middle cerebral artery; ACA: anterior cerebral artery; A1: A1 segment of the anterior cerebral artery; P1: P1 segment of the posterior cerebral artery; A2: A2 segment of the anterior cerebral artery.

Study	Study Design	Country	Year(s) of Study	Occlusion Site(s)	Alteplase Dosage (mg per kg)
Yang P. et al. [45]	RCT	China	2019	ICA, M1 and M2	0.9
Zi W. et al. [44]	RCT	China	2018–2020	ICA and M1	0.9
Suzuki K. et al. [46]	RCT	Japan	2017–2019	ICA and M1	0.6
LeCouffe N. E. et al. [47]	RCT	Netherlands, France and Belgium	2020	ICA, M1 and M2	0.9
Broeg–Morvay A. et al. [42]	Retrospective observational	Switzerland	2009–2014	ICA, M1 and M2	0.9 or 0.6
Bellwald S. et al. [35]	Retrospective observational	Switzerland, Germany	2009–2014	ICA, M1 and M2	0.9 or 0.6
Gong L. et al. [56]	Retrospective observational	China	2015–2018	MCA	No data
Weber R. et al. [41]	Retrospective observational	Germany	2012–2013	ICA, M1, M2, carotid T, basilar, A1, P1 and vertebral artery	0.9 or 0.6
Cappellari M. et al. [57]	Prospective cohort	Italy	2011–2017	ICA, M1 and M2	No data
Du M. et al [53]	Retrospective observational	China	2015–2018	Carotid T	0.9
Pienimäki J. P. et al. [58]	Retrospective observational	Finland	2016–2019	ICA and M1	0.9
Tong X. et al. [55]	Prospective observational	China	2017–2019	ICA, M1, M2, A1, A2, vertebrobasilar and P1	0.9
Wang H. et al. [59]	Retrospective observational	China	2014–2016	ICA, M1, M2 and A1	0.9

**Table 2:** Baseline characteristics of the study population. dMT, direct mechanical thrombectomy; BT, bridging therapy; DM, diabetes mellitus; HTN, hypertension; AF, atrial fibrillation, HLP, hyperlipidemia; ASA, acetylsalicylic acid; NDA, no data available.

Study	Number of Patients		Male %	Median Age (Years)	Onset NIHSS (Median)	DM	HTN	AF	Smoking	Cardiac Disease	HLP	Anticoagulant Use	ASA/Antiplatelet Use	Prior Stroke
Yang P. et al. [45]	dMT	327	57.8	69	17	59	193	152	73	24	13	28	48	43
	BT	329	55	69	17	65	201	149	68	17	14	36	56	47
Zi W. et al. [44]	dMT	116	56.9	70	16	25	69	62	28	30	18	NDA	NDA	14
	BT	118	55.9	70	16	20	74	62	29	19	22	NDA	NDA	19
Suzuki K. et al. [46]	dMT	101	55	74	19	16	61	57	42	7	30	19	16	12
	BT	103	70	76	17	17	61	64	54	7	37	17	18	14
LeCouffe N. E. et al. [47]	dMT	273	59	72	16	40	121/273	86	73/263	15	79	15	94	47
	BT	266	54	69	16	50	139/265	63	66/260	15	73	20	96	43
Broeg-Morvay A. et al. [42]	dMT	40	62.5	77 *	17	5	30	19	33	12	30	NDA	NDA	NDA
	BT	156	52.6	73 *	15	28	100	61	8	24	83	NDA	NDA	NDA
Bellwald S. et al. [35]	dMT	103	55.9	75 *	16	† 17/108	† 85/111	† 47/101	† 21/104	† 29/107	† 46/108	NDA	NDA	NDA
	BT	103	52.4	75 *	16	† 42/246	† 171/246	† 85/227	† 47/214	† 40/241	† 98/242	NDA	NDA	NDA
Gong L. et al. [56]	dMT	21	52	71 *	15	5	13	19	NDA	NDA	NDA	NDA	NDA	NDA
	BT	21	57	70 *	14	4	15	19	NDA	NDA	NDA	NDA	NDA	NDA
Weber R. et al. [41]	dMT	70	38	70.7	15	11	49	25	12	NDA	31	NDA	NDA	34
	BT	105	52	70.2	15.5	16	82	26	15	NDA	17	NDA	NDA	13
Cappellari M. et al. [57]	dMT	212	47	70.4 *	16 & 19 ‡	31	116	52	NDA	NDA	NDA	11	82	12
	BT	371	45	70.3 *	17 & 18 ‡	38	218	76	NDA	NDA	NDA	12	123	13
Du M. et al. [53]	dMT	57	32	66.9 *	18	12	33	33	15	17	NDA	10	39	7
	BT	54	28	65.2 *	18	7	28	31	10	10	NDA	9	39	5
Pienimaki J et al. [58]	dMT	48	62	72 *	14	11	31	19	NDA	7	NDA	NDA	NDA	NDA
	BT	58	64	69 *	16.5	13	29	37	NDA	12	NDA	NDA	NDA	NDA
Tong X. et al. [55]	dMT	394	64.7	65	17	67	214	124	165	76	26	7	56	76
	BT	394	62.7	65	16	63	214	124	166	63	31	4	60	63
Wang H. et al. [59]	dMT	138	76	67	16	† 38/203	† 130/203	† 92/203	† 49/203	† 557/203	NDA	9	NDA	15
	BT	138	78	67	17	† 27/160	† 99/160	† 75/160	† 43/160	† 32/160	NDA	0	NDA	16

* These are mean ages. † We have considered the matched group but some of the characteristics were only reported for all patients. ‡ The considered group has been divided into two subgroups and these two numbers are medians of the subgroups.

## Data Availability

The data that support the findings of this study are available from the corresponding author upon reasonable request.

## Supplementary Materials

The following supporting information can be downloaded at: https://www.mdpi.com/article/10.3390/life13010185/s1, Figure S1: Risk of bias of included RCTs, Figure S2: Risk of bias of included observational studies, Figure S3: Drapery plot of comparison of sICH between direct mechanical thrombectomy and bridge therapy in the anterior group, Figure S4: Drapery plot of comparison of mortality at 90 days between direct mechanical thrombectomy and bridge therapy in the anterior group, Figure S5: Drapery plot of comparison of good functional outcome at 90 days between direct mechanical thrombectomy and bridge therapy in the anterior group, Figure S6: Drapery plot of comparison of successful reperfusion rate between direct mechanical thrombectomy and bridge therapy in the anterior group, Figure S7: Drapery plot of comparison of sICH between direct mechanical thrombectomy and bridge therapy in the combined group, Figure S8: Drapery plot of comparison of mortality at 90 days between direct mechanical thrombectomy and bridge therapy in the combined group, Figure S9: Drapery plot of comparison of good functional outcome at 90 days between direct mechanical thrombectomy and bridge therapy in the combined group, Figure S10: Drapery plot of comparison of successful reperfusion rate between direct mechanical thrombectomy and bridge therapy in the combined group, Figure S11: Funnel plots of comparison of sICH rate between direct mechanical thrombectomy and bridge therapy in the combined and anterior groups, Figure S12: Funnel plots of comparison of mortality at 90 days between direct mechanical thrombectomy and bridge therapy in the combined and anterior groups, Figure S13: Funnel plots of comparison of good functional outcome at 90 days between direct mechanical thrombectomy and bridge therapy in the combined and anterior groups, Figure S14: Funnel plots of comparison of successful reperfusion rate between direct mechanical thrombectomy and bridge therapy in the combined and anterior groups, Table S1: Search strategies. Reference [89] is cited in the supplementary materials.
