# Supplementary material for: Direct Mechanical Thrombectomy Versus Prior Bridging Intravenous Thrombolysis in Acute Ischemic Stroke: A Systematic Review and Meta-Analysis
Source: Life (Basel). 2023 Jan 9;13(1):185. doi: 10.3390/life13010185 (PMC9863165; doi:10.3390/life13010185)

Data Supplement

Table S1. Search strategies

Database	Search Algorithm
PubMed, EMBASE, Scopus, Cochrane	1. exp Mechanical Thrombolysis/<br>2. ((mechanical or endovascular) adj2 (treat* or thromb* or (clot adj2 disrupt*))).ti,ab.<br>3. exp Stents/<br>4. (stent* or Trevo or Merci or Penumbra).ti,ab.<br>5. 1 or 2 or 3 or 4<br>6. exp Stroke/<br>7. ((brain or cerebr* or subcortical or hemispher* or arter* or lacunar) adj2 infarct*).ti,ab.<br>8. (stroke* or apoplex* or ((brain or cerebrovascular) adj2 (attack or injury or accident* or insult*)) or CVA).ti,ab.<br>9. 6 or 7 or 8<br>10. exp Brain Ischemia/<br>11. ((brain or cerebral or hypoxia) adj2 ischem*).ti,ab.<br>12. (ischem* adj2 (encephalopath* or attack* or stroke*)).ti,ab.<br>13. 10 or 11 or 12<br>14. exp Thrombolytic Therapy/

	15. ((throm* or fibrinolytic) adj2 therap*).ti,ab.<br>16. exp Fibrinolysis/<br>17. fibrinoly*.ti,ab.<br>18. exp Plasminogen Activators/<br>19. (plasminogen adj2 activat*).ti,ab.<br>20. (bridg* or anistreplase or alteplase or tPA or rt-PA).ti,ab.<br>21. exp Antithrombins/<br>22. (antithrombin* or (thrombin adj2 inhibitor*)).ti,ab.<br>23. 14 or 15 or 16 or 17 or 18 or 19 or 20 or 21 or 22<br>24. exp Hemorrhage/<br>25. (hemorrhag* or bleed*).ti,ab.<br>26. exp Long Term Adverse Effects/<br>27. ((adverse or side or undesir* or negat*) adj2 (impact* or effect* or reaction* or event* or outcome*)).ti,ab.<br>28. exp Mortality/<br>29. (mortalit* or ((death or fatal*) adj2 rate*)).ti,ab.<br>30. exp Safety/<br>31. (safe* or tolerab* or inciden* or predict* or prognos* or course*).ti,ab.<br>32. 24 or 25 or 26 or 27 or 28 or 29 or 30 or 31<br>33. 5 and 9 and 13 and 23 and 32

Figure S1. Risk of bias of included RCTs.

Risk of Bias Domain / Study	The Randomization Process	Deviations From Intended Interventions	Missing Outcome Data	Measurement of The Outcome	Selection of The Reported Result	Overall Risk of Bias
Yang P. et al. (2020)	+	×	+	+	+	×
Zi W. et al. (2021)	+	+	+	+	+	+
Suzuki K. et al. (2021)	+	?	+	+	+	?
LeCouffe N. E. et al. (2021)	+	+	+	+	+	+

+ Low Risk

? Some Concerns

× High Risk

Figure S2. Risk of bias of included observational studies.

Risk of Bias Domain / Study	Confounding	Selection of Participants Into the Study	Deviations From Intended Interventions	Classification of Interventions	Missing Data	Measurement of Outcomes	Selection of The Reported Result	Overall Risk of Bias
Broeg-Morvay A. et al. (2016)	−	+	+	+	+	+	+	+
Gong L. et al. (2019)	−	+	+	+	+	−	−	−
Weber R. et al. (2017)	−	+	+	+	+	−	−	−
Wang H. et al. (2017)	−	+	+	+	+	+	+	+
Bellwald S. et al. (2017)	+	+	+	+	+	+	+	+
Cappellari M. et al. (2021)	−	+	+	+	+	−	−	−
Du M. (2021)	+	+	+	+	+	−	−	+
Pienimäki J. P. et al. (2021)	−	+	+	+	+	+	+	+
Tong X. et al. (2021)	+	+	+	+	−	−	−	−

+ Low Risk

− Moderate Risk

× Serious Risk

Figure S3. Drapery plot of comparison of sICH between direct mechanical thrombectomy and bridge therapy in the anterior group.

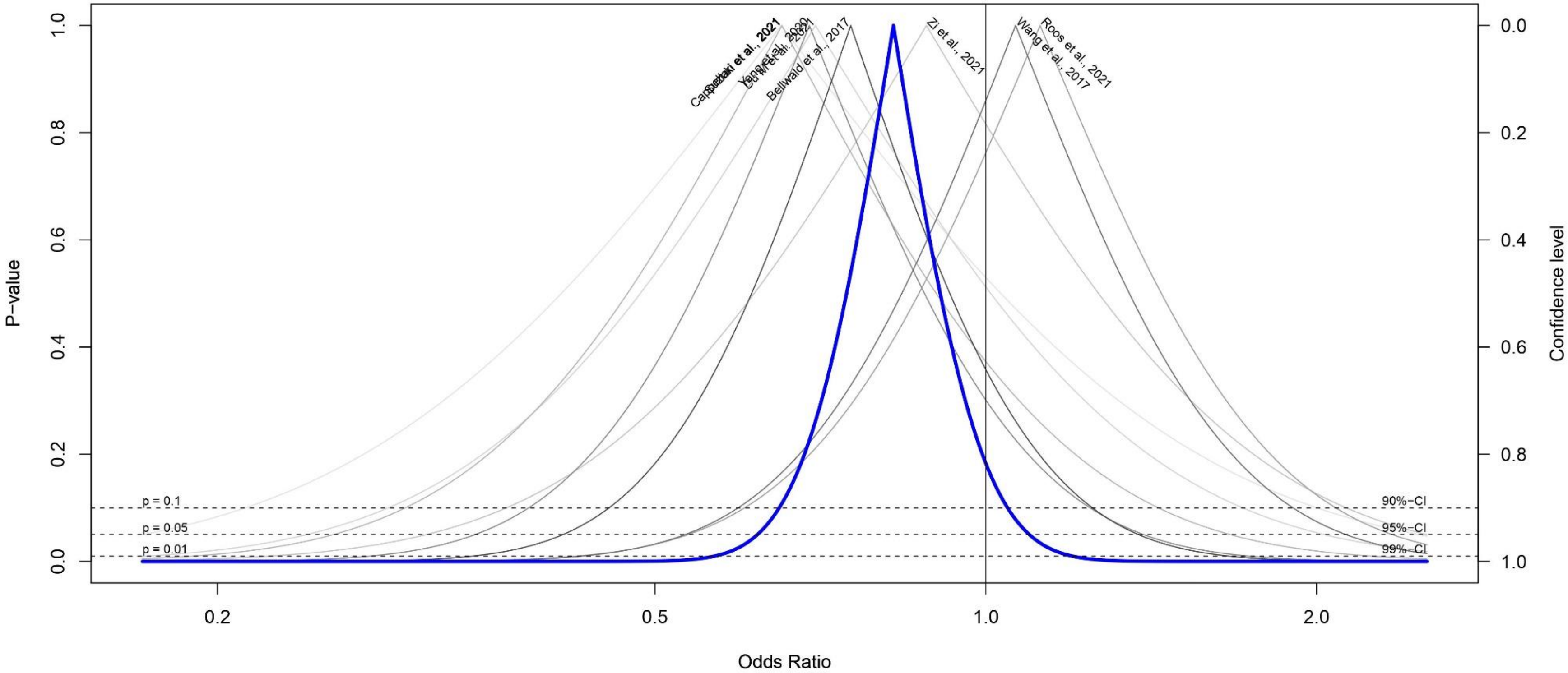

Figure S4. Drapery plot of comparison of mortality at 90 days between direct mechanical thrombectomy and bridge therapy in the anterior group.

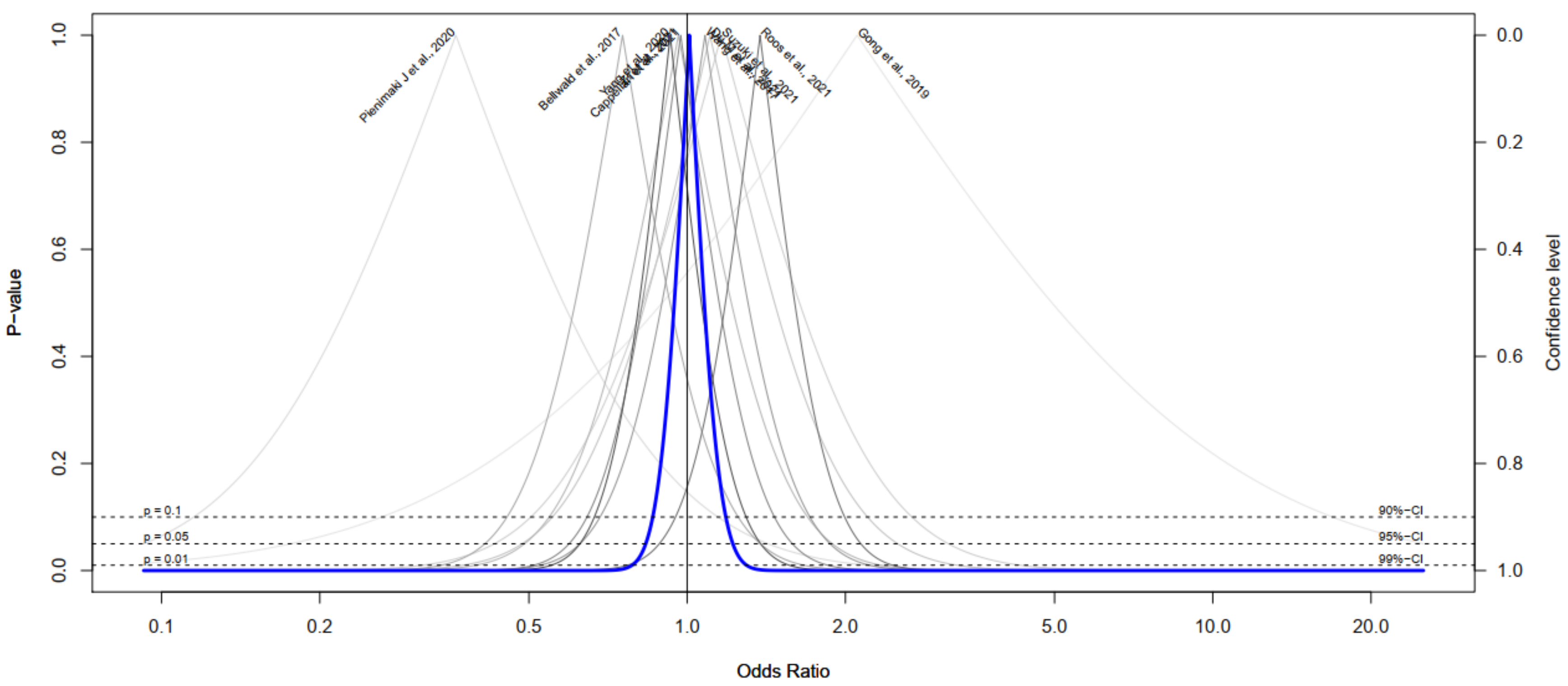

Figure S5. Drapery plot of comparison of good functional outcome at 90 days between direct mechanical thrombectomy and bridge therapy in the anterior group.

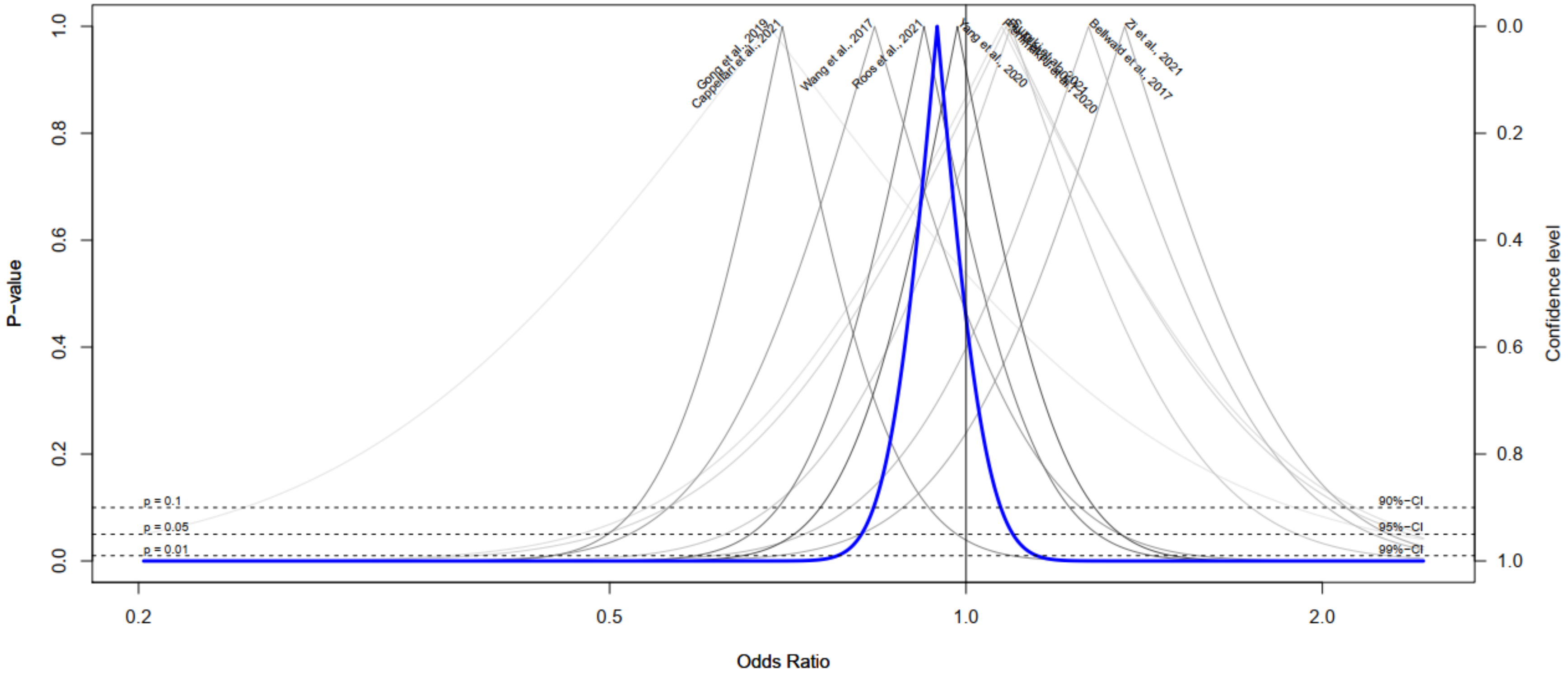

Figure S6. Drapery plot of comparison of successful reperfusion rate between direct mechanical thrombectomy and bridge therapy in the anterior group.

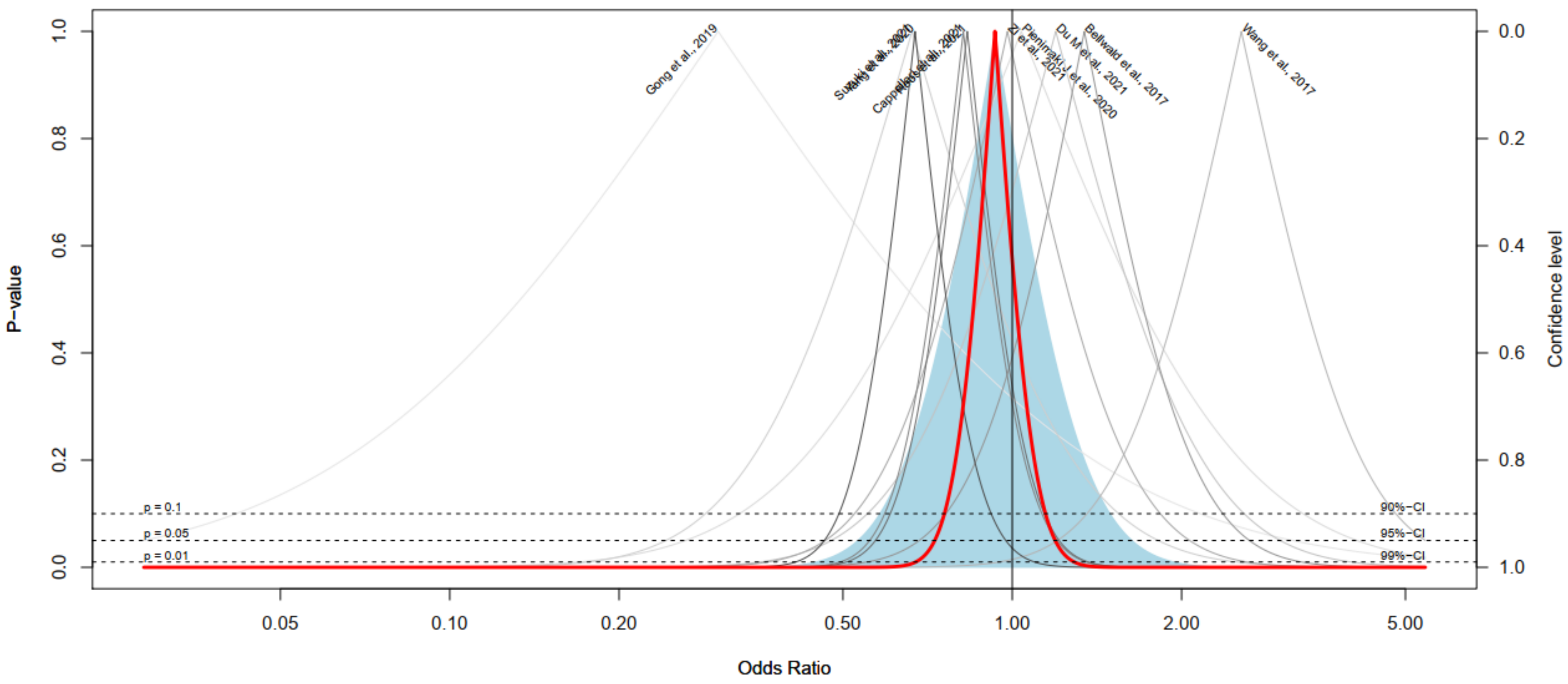

Figure S7. Drapery plot of comparison of sICH between direct mechanical thrombectomy and bridge therapy in the combined group.

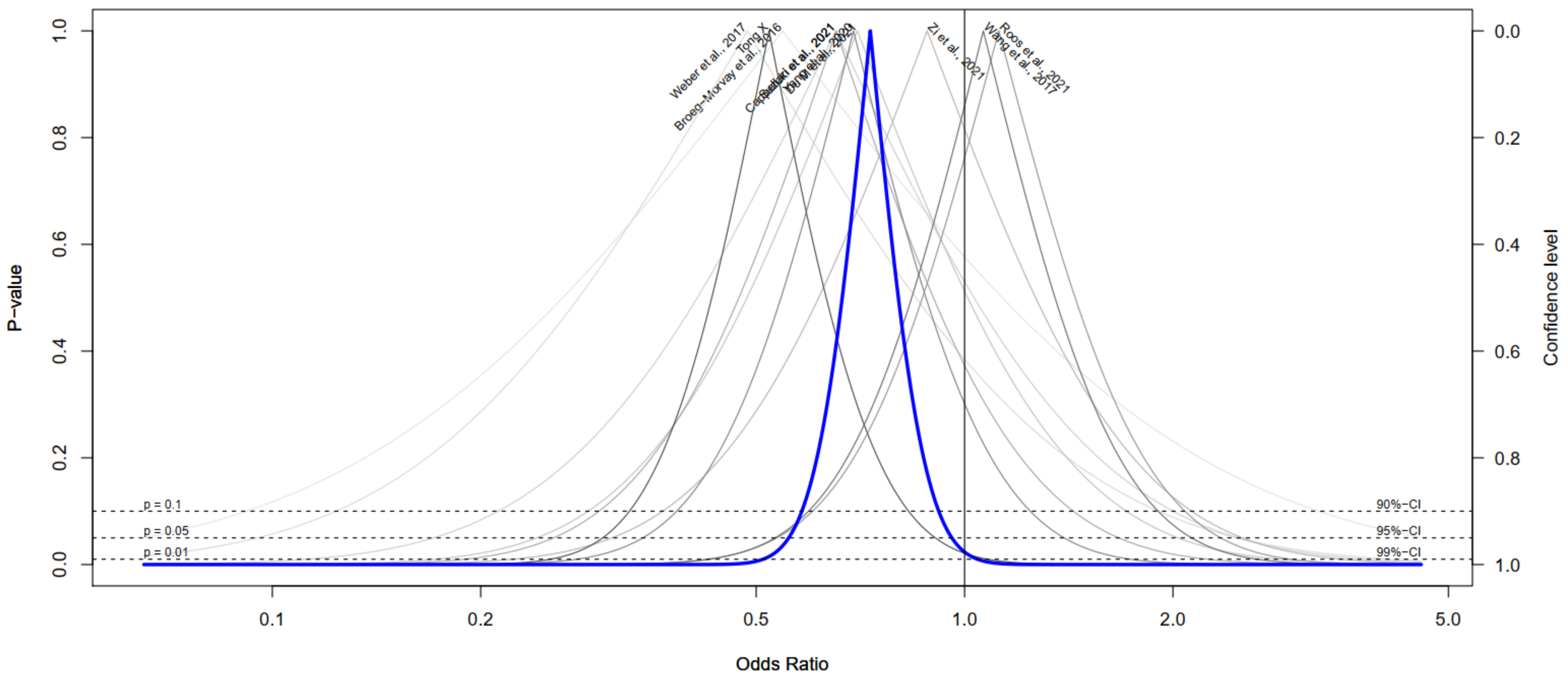

Figure S8. Drapery plot of comparison of mortality at 90 days between direct mechanical thrombectomy and bridge therapy in the combined group.

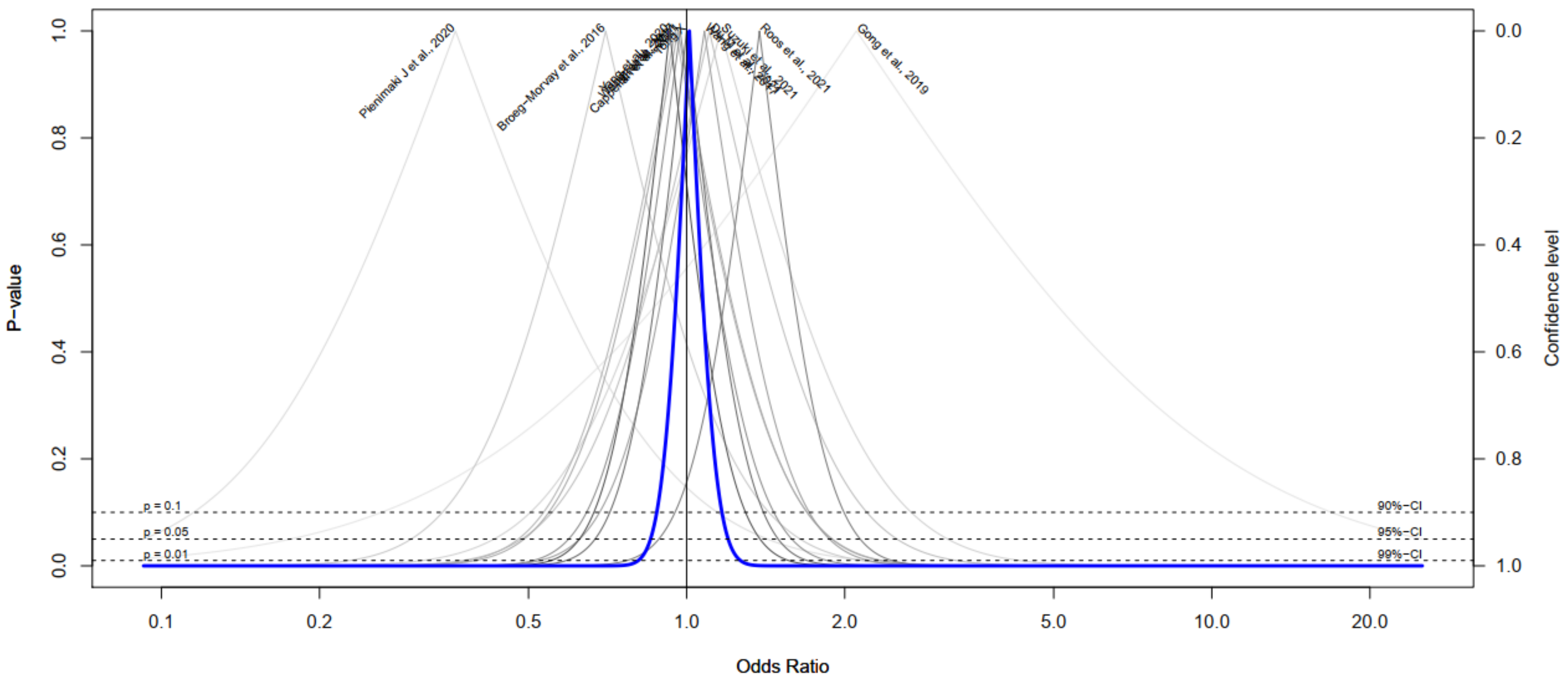

Figure S9. Drapery plot of comparison of good functional outcome at 90 days between direct mechanical thrombectomy and bridge therapy in the combined group.

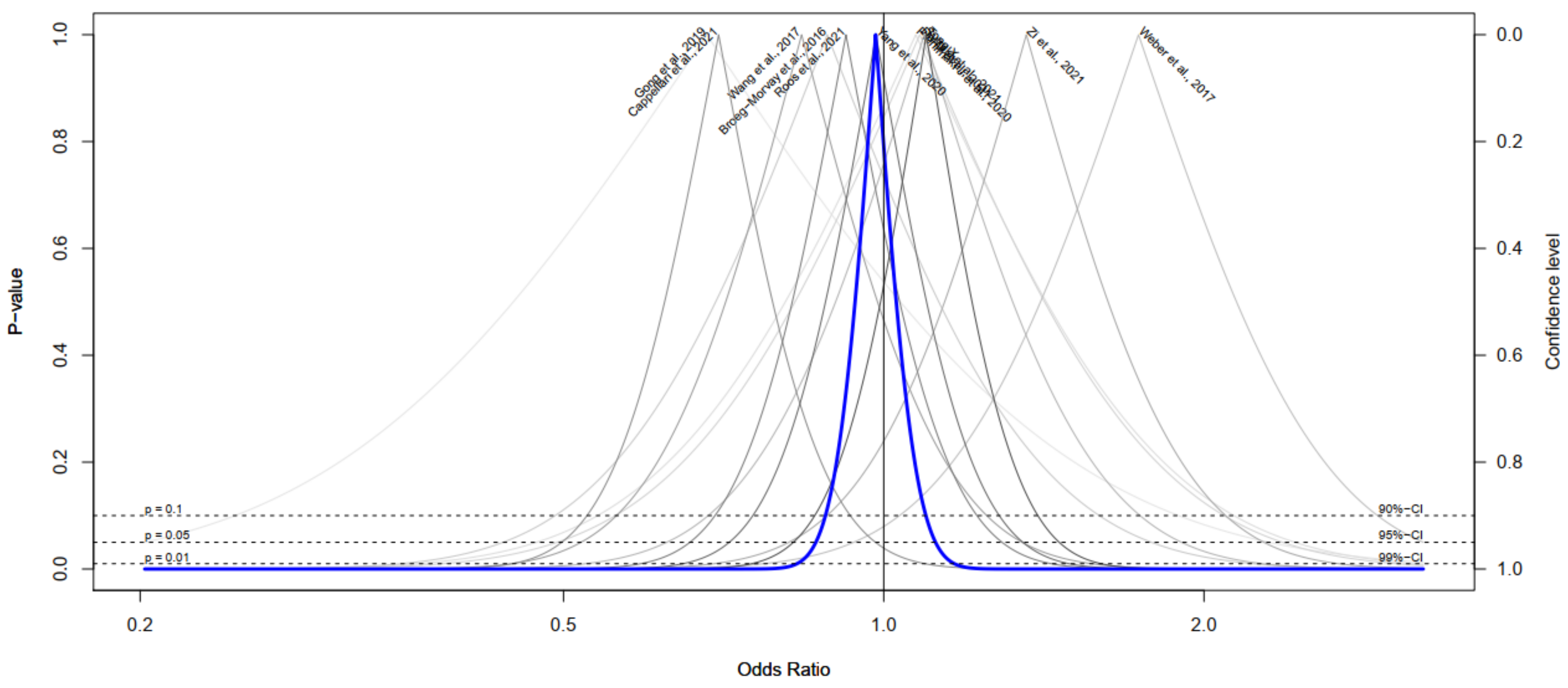

Figure S10. Drapery plot of comparison of successful reperfusion rate between direct mechanical thrombectomy and bridge therapy in the combined group.

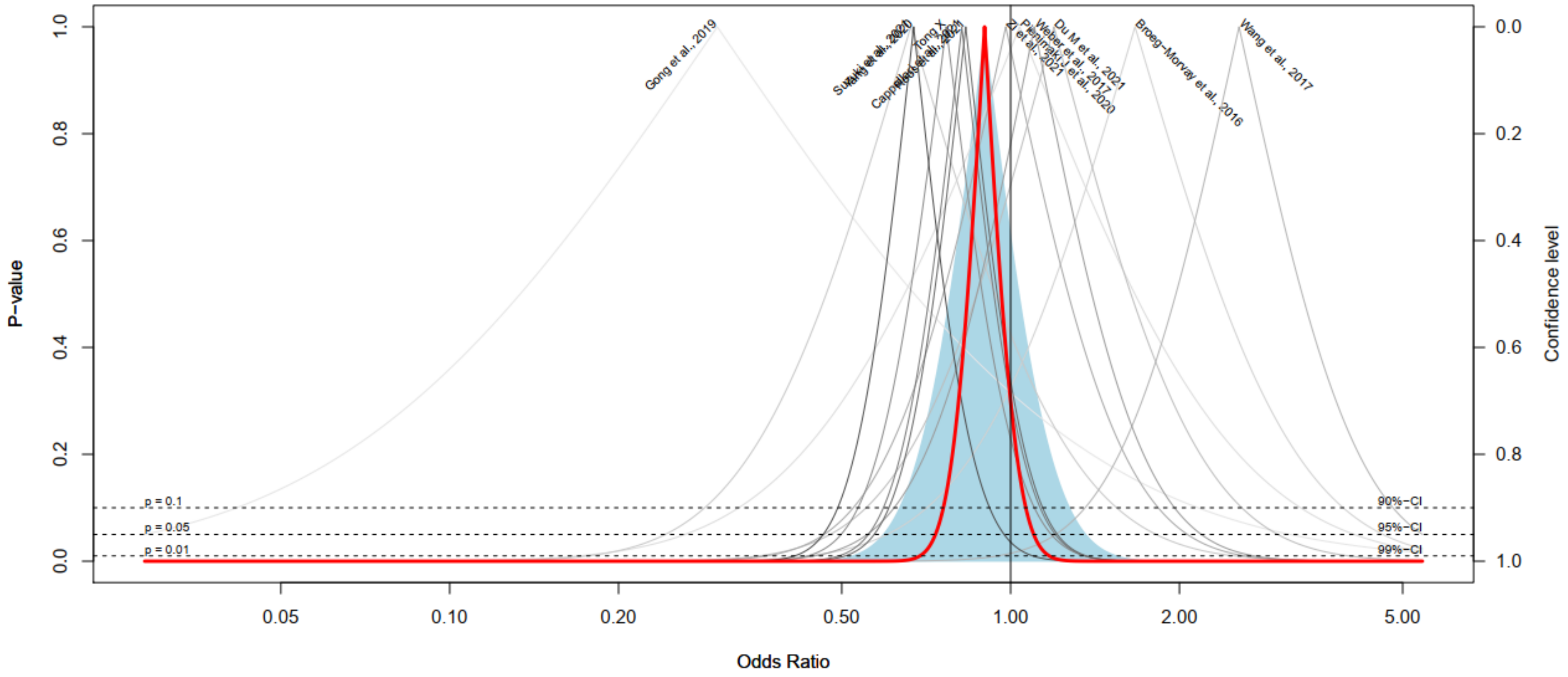

Figure S11. Funnel plots of comparison of sICH rate between direct mechanical thrombectomy and bridge therapy in the combined and anterior groups.

sICH (anterior)

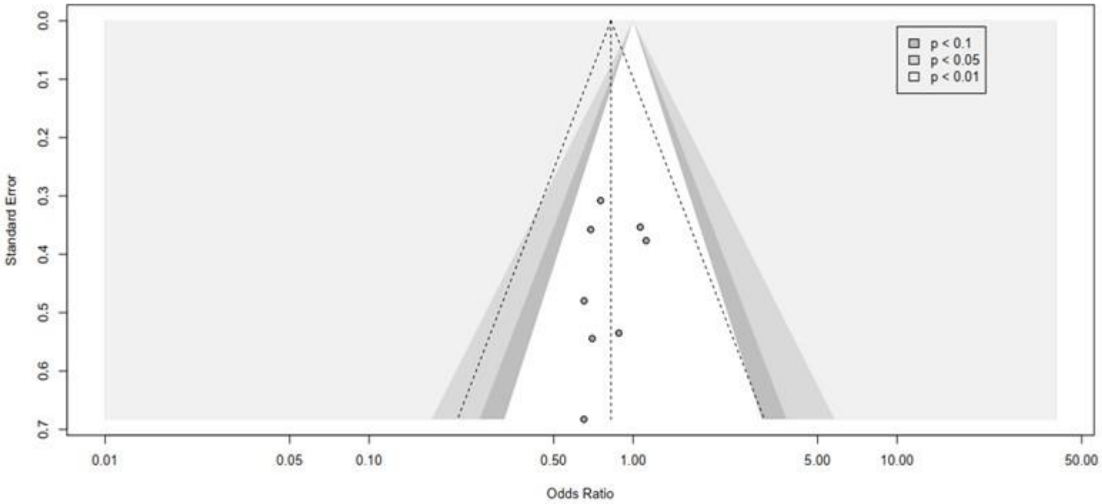

sICH (combined)

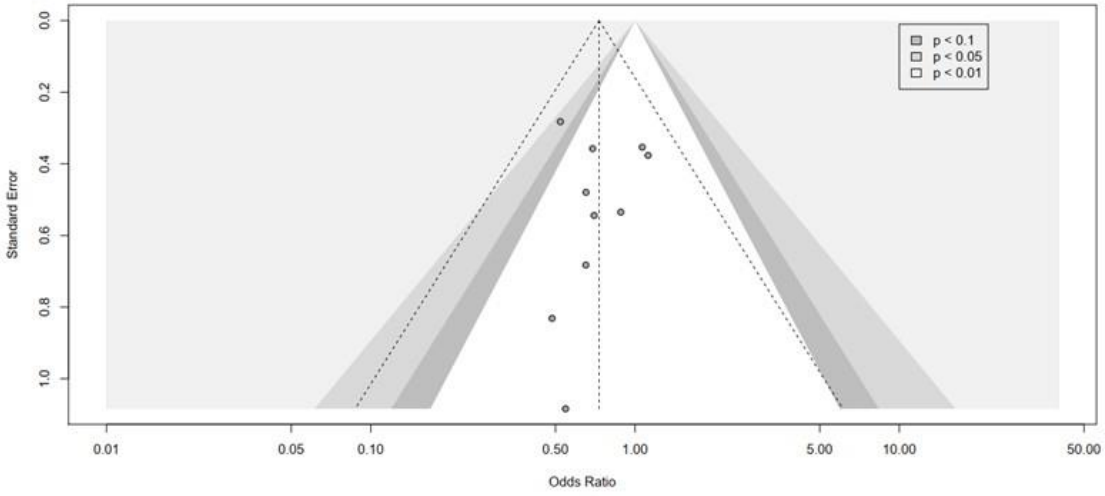

Figure S12. Funnel plots of comparison of mortality at 90 days between direct mechanical thrombectomy and bridge therapy in the combined and anterior groups.

Mortality (anterior)

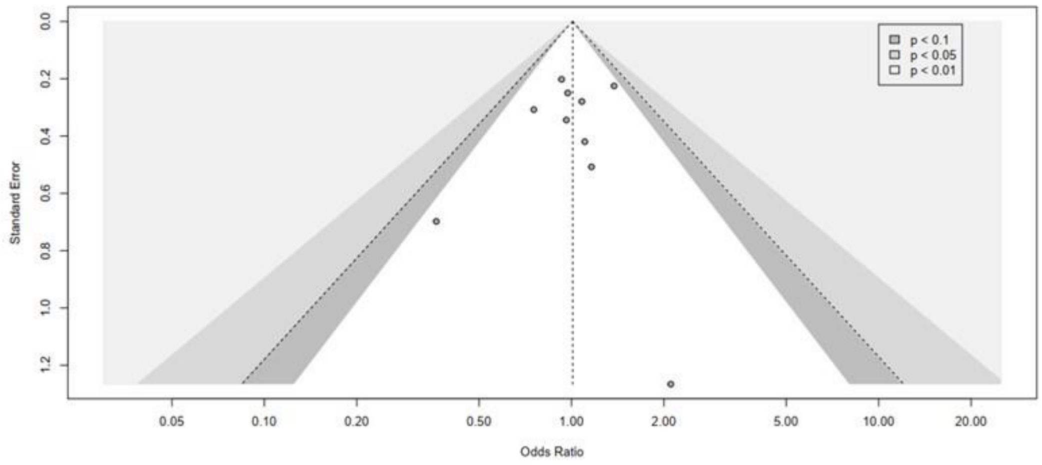

Mortality (combined)

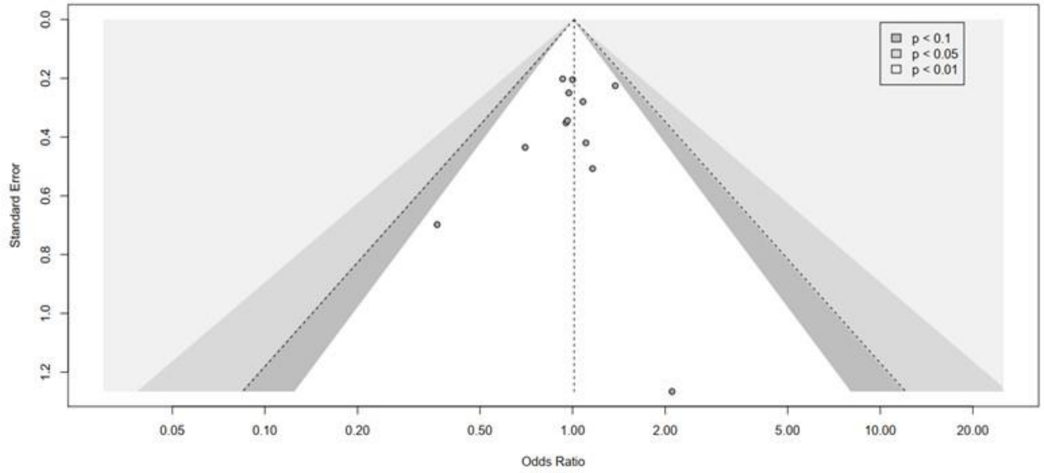

Figure S13. Funnel plots of comparison of good functional outcome at 90 days between direct mechanical thrombectomy and bridge therapy in the combined and anterior groups.

Good functional outcome (anterior)

Good functional outcome (combined)

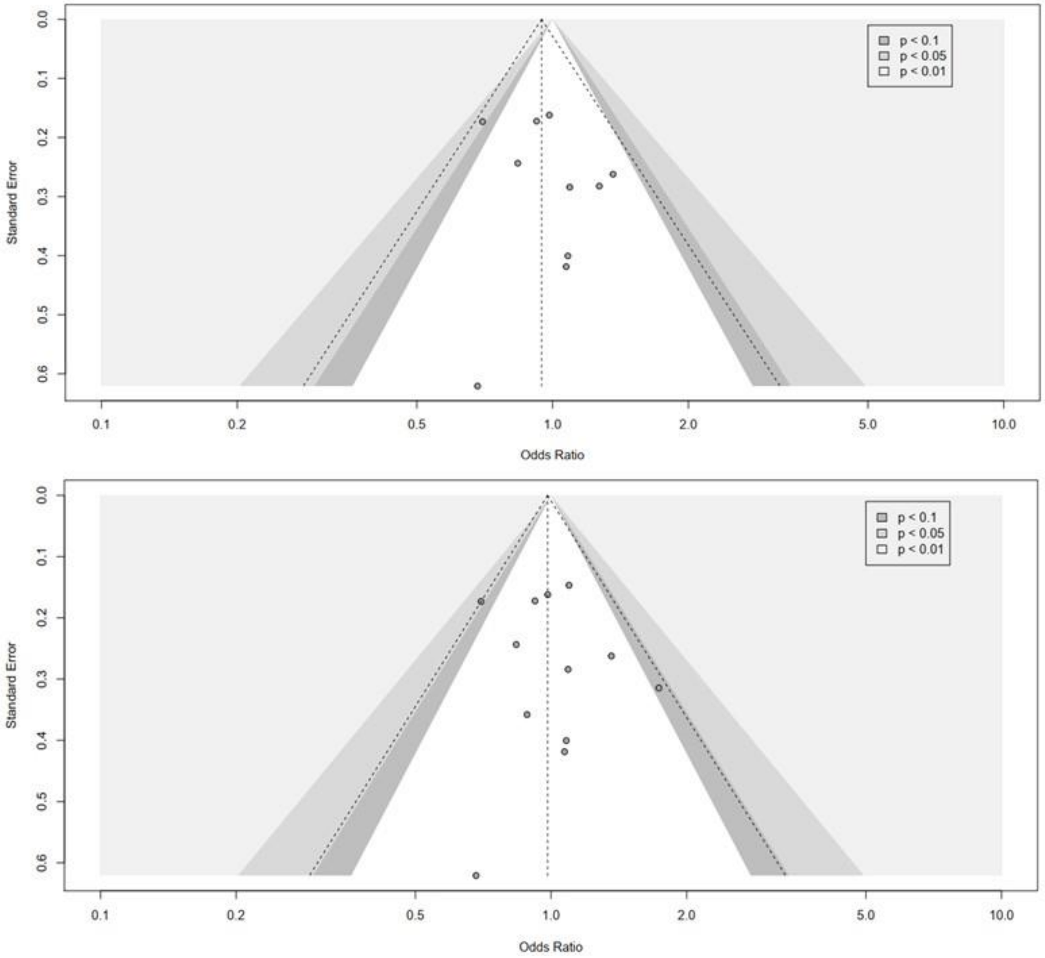

Figure S14. Funnel plots of comparison of successful reperfusion rate between direct mechanical thrombectomy and bridge therapy in the combined and anterior groups.

Successful reperfusion rate (anterior)

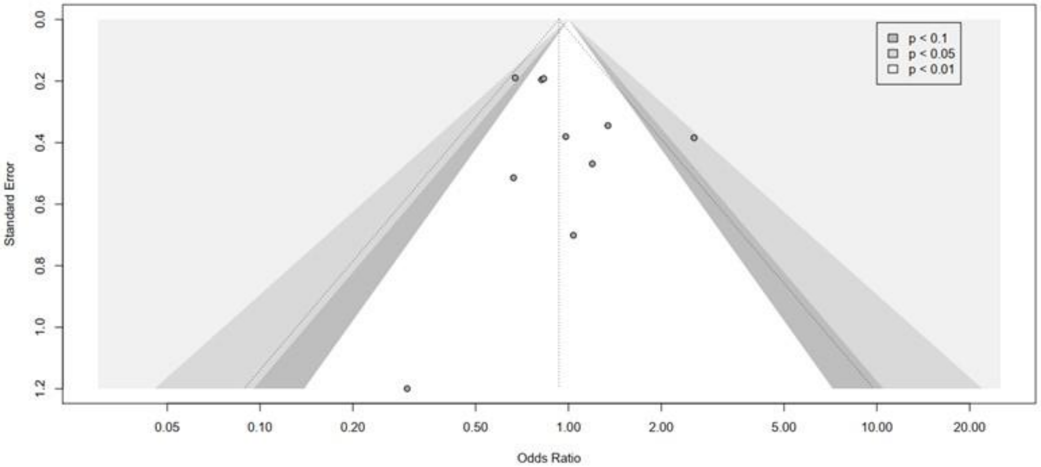

Successful reperfusion rate (combined)

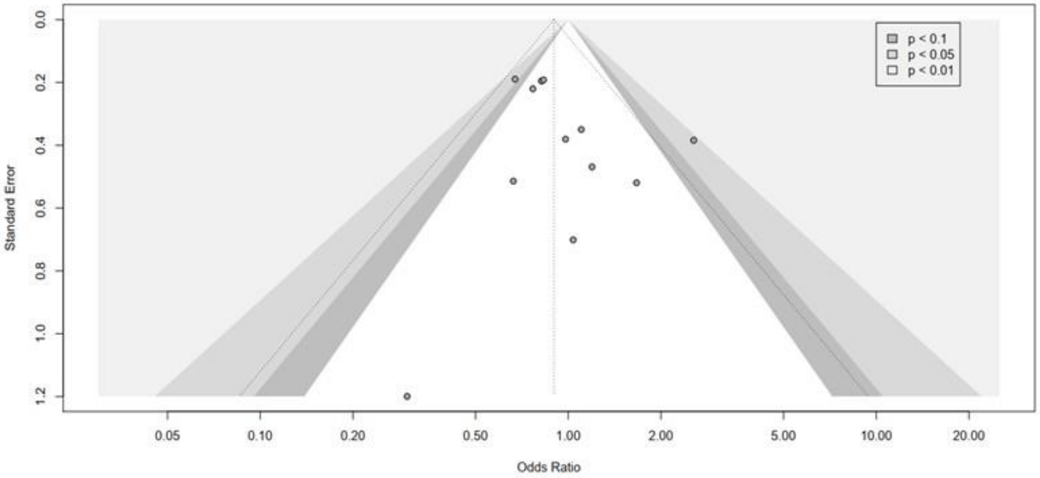

Supplement: Supplementary file 1 [file life-13-00185-s001.zip › Data Supplement.pdf]
